# Serological Evidence of Infectious Laryngotracheitis Infection and Associated Risk Factors in Chickens in Northwestern Ethiopia

**DOI:** 10.1155/2022/6096981

**Published:** 2022-08-08

**Authors:** Mastewal Birhan, Ashenafi Syoum, Saddam Mohammed Ibrahim, Tewodros Fentahun, Addisu Mohammed, Nega Berhane, Molalegne Bitew, Esayas Gelaye, Malede Birhan Atanaw, Belayneh Getachew, Bereket Dessalegn, Anmaw Shite Abat, Kassahun Berrie, Kassaye Adamu, Takele Abayneh

**Affiliations:** ^1^Institute of Biotechnology, University of Gondar, Gondar, Ethiopia; ^2^College of Veterinary Medicine and Animals Sciences, University of Gondar, Gondar, Ethiopia; ^3^College of Veterinary Medicine, Samara University, Semera, Ethiopia; ^4^Bio and Emerging Technology Institute, Addis Ababa, Ethiopia; ^5^National Veterinary Institute, Bishoftu, Ethiopia

## Abstract

Infectious laryngotracheitis (ILT) is a disease of high economic consequence to the poultry sector. Gallid herpesvirus 1 (GaHV-1), a.k.a infectious laryngotracheitis virus (ILTV), under the genus *Iltovirus,* and the family Herpesviridae, is the agent responsible for the disease. Despite the clinical signs on the field suggestive of ILT, it has long been considered nonexistent and a disease of no concern in Ethiopia. A cross-sectional study was conducted from November 2020 to June 2021 in three selected zones of the Amhara region (Central Gondar, South Gondar, and West Gojjam zones), Ethiopia, with the objective of estimating the seroprevalence of ILTV in chickens and identifying and quantifying associated risk factors. A total of 768 serum samples were collected using multistage cluster sampling and assayed for anti-ILTV antibodies using indirect ELISA. A questionnaire survey was used to identify the potential risk factors. Of the 768 samples, 454 (59.1%, 95% CI: 0.56–0.63) tested positive for anti-ILTV antibodies. Mixed-effect logistic regression analysis of potential risk factors showed that local breeds of chicken were less likely to be seropositive than exotic breeds (OR: 0.38, 95% CI: 0.24–0.61). In addition, factors such as using local feed source (OR: 6.53, 95% CI: 1.77–24.04), rearing chickens extensively (OR: 1.97, 95% CI: 0.78–5.02), mixing of different batches of chicken (OR: 14.51, 95% CI: 3.35–62.77), careless disposal of litter (OR: 1.62, 95% CI: 0.49–4.37), lack of house disinfection (OR: 11.05, 95% CI: 4.09–47.95), lack of farm protective footwear and clothing (OR: 20.85, 95% CI: 5.40–80.45), and careless disposal of dead chicken bodies had all been associated with increased seropositivity to ILTV. Therefore, implementation of biosecurity measures is highly recommended to control and prevent the spread of ILTV. Furthermore, molecular confirmation and characterization of the virus from ILT suggestive cases should be considered to justify the use of ILT vaccines.

## 1. Introduction

Poultry farming is one of the rapidly emerging sectors with a key role in global food security [1]. In Ethiopia, chicken production is widely spread with almost every rural family rearing chickens as a valuable source of family protein and income [2]. As of the 2021 report, the country's chicken population was estimated to be 57 million [3]. Nevertheless, this huge potential is unable to satisfy the growing domestic demand for chicken products, and the economic contribution of the sector remained marginal for various reasons [4].

Owing to the rapid population growth and change in living standards, the demand for chicken meat and eggs in Ethiopia is expected to rise by 268% and 737%, respectively, between 2012 and 2050 [5]. As a coping strategy, the Ethiopian government has outlined policies for intensifying the poultry production system by introducing exotic breeds and advanced technologies [6]. As a result, many government-owned multiplication and distribution centers along with nongovernmental organizations have been importing and distributing exotic breeds to augment the intensification process [7]. However, there was a growing concern of introduction of diseases of various etiologies into the poultry system concurrent with the importation of exotic breeds [8].

Among the infectious diseases of potential damage to the poultry sector is infectious laryngotracheitis (ILT). Infectious laryngotracheitis is a highly contagious respiratory disease of chickens caused by ILT virus (ILTV), a.k.a Gallid alphaherpesvirus 1 (GaHV-1), which belongs to the genus *Iltovirus*, subfamily Alphaherpesvirinae, and the family Herpesviridae [9]. The sever form of the disease results in respiratory depression, gasping, and expectoration of bloody exudates with high rates of morbidity and mortality up to 70% in an acute form of infection [10].

Vaccination and biosecurity are the main strategies for the control of the disease. Live attenuated and recombinant viral vector vaccines are commercially available for use against ILT. Live attenuated vaccines (LAVs) were developed by multiple passages in embryonated eggs recognized as chicken embryo origin (CEO) [11], or by multiple passages in tissue culture recognized as tissue culture origin (TCO) [12]. Despite their high effectiveness, GaHV-1 LAVs, particularly the CEO vaccines, carry a high risk of reversion to virulence [13] resulting in outbreaks [14]. The recombinant viral vector vaccines were developed as safest alternatives to the LAVs. Currently, fowl-pox virus (FPV) and herpesvirus of turkeys (HVT) based GaHV-1 vectored vaccines expressing immunogenic proteins of ILTV are widely available on market. Experimental evidence had shown that GaHV-1 viral vector vaccines improve birds' performance and reduce clinical signs of the disease but are not as effective as CEO and TCO vaccines in diminishing shedding of the challenge virus [15, 16]. Hence, combination strategies of both the recombinant and live ILT vaccines have been practiced in some multiage layer and heavyweight broiler complexes successfully for optimal outcomes [17]. Even though the Ethiopian government has not officially endorsed the introduction of ILT vaccines yet, the CEO and TCO versions of the vaccines have reportedly been used by some private poultry farms.

In spite of the economic implications of the disease and its high contagiousness, there are limited scientific reports on the status of ILTV in Ethiopia. However, the evident clinical signs on the field suggestive of the disease and the increased demand of the government to commercialize the poultry production have invoked a national need to identify the disease urgently and plan an appropriate intervention. Accordingly, in recent years, Tesfaye et al. [18] and Roba et al. [19] reported serological evidence of ILTV infection in Central and South Ethiopia (19.4%) and Ada'a district in Oromia region (54.7%), respectively. The present study took place in the Amhara region (estimated to have 19 million of chickens) [3] with the objective of assessing serum anti-ILTV antibodies and the associated risk factors in chickens, and it was the first of its kind in the region.

## 2. Materials and Methods

### 2.1. Study Area

**Figure 1:**
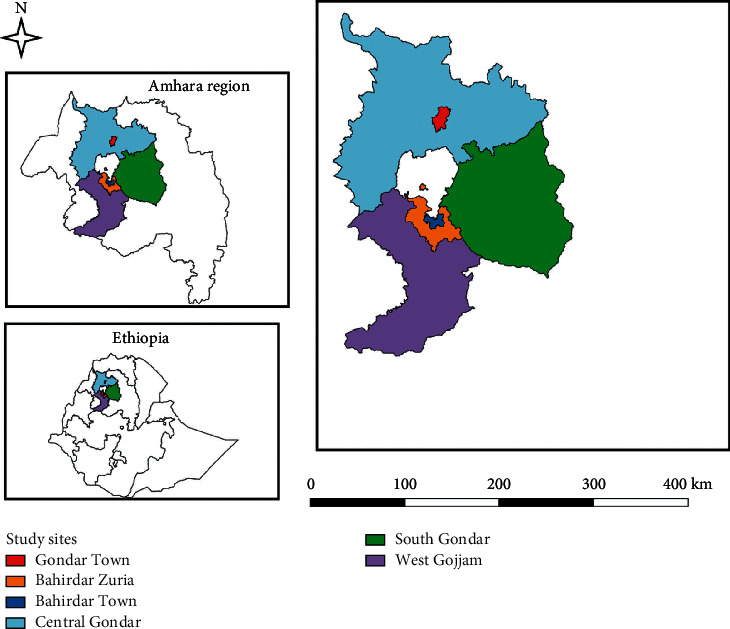
Map showing the study area (created using QGIS 3.18).

This study was conducted in three administrative zones of the Amhara National Regional State, located in the Northwestern part of Ethiopia. Study zones included were Central Gondar, West Gojjam, and South Gondar (Figure 1). Central Gondar has an altitude ranging between 1750 and 2600 m above sea level, and it is found just to the North of Lake Tana. It has a total annual rainfall of 1047.6 mm, mean maximum temperature of 27.4°C, and mean minimum temperature of 14.7°C and relative humidity of 45% [20]. The total chicken flock in the study zone was estimated to be 3,244,120 [3].

West Gojjam zone is situated at 11° 09′ 60.00″ N latitude and 37° 14′ 60.00″ E longitude with an altitude ranging from 1500 to 3420 m above the sea level [21]. The mean annual temperature ranges from 22°C to 27°C in the lowlands and between 10°C and 22°C in the highlands. The long-term mean annual rainfall is 1165.2 mm. However, areas in the specific study sites received 1100 to 1360 mm of mean annual rainfall per year [22]. The total chicken flock in the study zone was estimated to be 3,729,350 [3].

Geographically, South Gondar zone is located between 11° 02′–12° 33′ N latitude and 37° 25′–38° 43′ E longitude. This zone is well known for diverse topography ranging from flat and low grazing land to high cold mountains. The altitude is 1500 to 3,600 m above the sea level and the average yearly rainfall varies from 700 mm to 1300 mm [23]. The total chicken flock in the study zone was estimated to be 1,885,633 [3].

### 2.2. Study Design and Study Population

A cross-sectional study design was applied from November 2020 to June 2021 to determine the seroprevalence of ILTV in chicken and the associated risk factors. The chickens included in the study were indigenous breeds and exotic Sasso T line (Ruby T) chickens, aged 3-week-old and above, with no history of vaccination, and that were kept both for meat and egg production. Despite the lack of confirmatory reports, respiratory diseases of similar nature with ILT have been reported by owners in the study sites.

### 2.3. Sampling Technique and Sample Size Estimation

Multistage cluster sampling technique was implemented to select the study zones, districts, kebeles/villages (Kebele: the smallest administrative units in Ethiopia), households/farms, and individual chickens for serum sampling. Of all the administrative zones of the Amhara region, three zones were selected based on the population of chickens they owned. Then, 7 districts were selected from those three zones, that is, 3 from Central Gondar, 2 from South Gondar, and 2 from West Gojjam using a simple random technique. Similarly, kebeles (*n* = 16), villages, households/farms, and individual chickens (*n* = 768) were all selected using a simple random sampling technique.

The sample size for this study was estimated using the formula provided by Bennett et al. [24] as follows:

(1) $$\begin{array}{c} n=gc=\frac{P\left(1–P\right)D}{{\text{SE}}^{2}}, \end{array}$$

where “*n*”: the sample size, “*p*”: the prevalence as a percentage, “*D*”: the design effect, “SE”: the standard error, “*g*”: the average number of individuals sampled per cluster, and “*c*”: the number of clusters.

(2) $$\begin{array}{c} D=1+\left(g-1\right)\text{ICC}. \end{array}$$

The estimate of intracluster correlation coefficient (ICC) for most infectious diseases does not exceed 0.2 [25]. So, considering 0.2 for the cluster (village) and the possibility of collecting about 34 serum samples per village (g), *D* equals 7.6. Sampling 34 animals per village with an expected prevalence of 50% (as no previous studies were conducted in the study area) and a standard error of 0.05 gave about 22 clusters, and thus a total sample size of 760. Therefore, a total of 768 blood samples werecollected in this study.

### 2.4. Blood Sample Collection and Serum Preparation

Whole blood sample (2-3 ml) was collected aseptically from wing vein (brachial vein) of each chicken using sterile 3 ml disposable syringes with 22-gauge × 1¼ inch needle. Blood was then immediately drained into plain vacutainer tubes. Then, the blood samples were kept in an icebox at approximately 45° inclination and transported to the Veterinary Microbiology laboratory of the University of Gondar. The blood samples were then allowed to clot in a slant position overnight at room temperature to allow for separation off the serum from the blood clot. Subsequently, the sera were poured off into sterile 1.5 ml Eppendorf tubes and transported in an icebox to the National Veterinary Institute (NVI) and kept at −20°C until serological analysis, for the presence of anti-ILTV antibodies. All necessary information related to each chicken including age, breed, sex, feeding status, farming type, production type, batch management methods, litter management, and protective footwear and clothing status was properly recorded on the data recording sheet.

### 2.5. Serum Analysis: Detection of Anti-ILTV Antibody in Chicken Serum

Each serum sample (after being diluted at the ratio, 1 *µ*l sample: 500 *µ*l of diluent) was tested for the presence of anti-ILTV antibodies using commercial indirect ELISA kit (IDvet Screen® ILT Indirect, 310 rue Louis Pasteur, 34790 Grabels, France) following the procedure provided by manufacturer. The optical densities (ODs) were read photometrically at a wavelength of 450 nm. Sample positivity or negativity was determined by calculating the sample (diluted sera) to positive (S/P) ratio according to the methods provided by the manufacturer as follows:

(3) $$\begin{array}{c} \frac{S}{P}=\frac{\text{ODS}-\text{ODNC}}{\text{ODPC}-\text{ODNC}}, \end{array}$$

where “S/P”: sample to positive ratio, “ODS”: optical density of a given sample, “ODNC”: optical density of the negative controls, “ODPC”: optical density of the positive controls.

Accordingly, sample to positive (S/P) ratios of ≤0.3 and > 0.3 were read as negative and positive, respectively.

### 2.6. Questionnaire Survey

A semistructured questionnaire was used to assess for potential risk factors of ILTV infection in chicken flocks. Epidemiological data such as age, breed, sex, feeding status and farming type, production type, batch management methods, litter management, carcass management, and status of protective footwear and clothing were considered potential factors.

### 2.7. Data Management and Statistical Analysis

Data obtained were entered into a Microsoft Excel spreadsheet (Microsoft Excel 2010, Microsoft Corporate, USA), coded, and then imported into STATA version 14 (Stata Corp., College Station, TX, USA) for statistical analysis. The data were summarized using descriptive statistics. Seroprevalence of ILTV was computed by dividing the total number of seropositive chickens by the total number examined. Mixed effect logistic regression analysis was used to identify association between potential risk factors and seropositivity. Univariable logistic regression analysis taking flock as a random effect was first performed, and factors with *p* < 0.25 were included in the multivariable logistic regression model. Associations were considered statistically significant when *p* < 0.05 at 95% confidence level. Odds ratio with a 95% confidence interval was used to express the strength of association.

## 3. Results

### 3.1. Summary of Potential Risk Factors

Table 1 shows summary of the potential risk factors recorded from the study sites and their respective frequencies. As shown, the proportional number of chickens (*n* = 256) was sampled among the three study zones. The highest number of samples was collected from local breed, female, adult, layer chickens. The majority of the study chickens were managed extensively in a well-ventilated environment. Most chicken keepers rely on commercially available feeds for their chickens. In terms of managing chickens, mixing of chickens of different batches was a common practice. The biosecurity data showed that the majority of chicken are managed in a highly risky environment where sanitation is not fully practiced, litters and carcass are disposed randomly, and protective footwear and clothing are not available.

### 3.2. Seroprevalence of ILTV

In this study, a total of 768 samples were tested for anti-ILTV antibodies using an indirect ELISA. The overall seroprevalence of ILTV was 59.1% (95% CI: 0.56–0.63) (454/768). The highest prevalence was observed in Central Gondar zone (78.1%, 95% CI: 0.73–0.83), followed by West Gojjam zone (58.6%, 95% CI: 0.52–0.65) and South Gondar zone (40.6%, 95% CI: 0.35–0.47) (Table 2).

### 3.3. Association of Potential Risk Factors with Seropositivity

#### 3.3.1. Intrinsic Risk Factors

Seroprevalence of ILTV in relation to host-specific (intrinsic) risk factors (breed, sex, age, and production purpose) was analyzed as the proportion of aﬀected chickens out of the total examined. The multivariable analysis showed that local breed chickens had a lower odd of seropositivity to ILTV as compared to exotic breeds (OR: 0.38, 95% CI: 0.24–0.61) (*p* ≤ 0.001) (Table 3).

#### 3.3.2. Extrinsic Risk Factors

Environmental (extrinsic) factors such as zones, farming type, feed source, chicken batch management method, house ventilation, house disinfection, litter management, protective footwear and clothing, and carcass management were evaluated as potential risk factors for the seroprevalence of ILTV. As indicated in Table 4, seroprevalence showed a statistically significant variation among study zones, with South Gondar (OR: 0.49, 95% CI: 0.29–0.86) and West Gojjam (OR: 0.59, 95% CI: 0.34–0.97) having a lower odd of seropositivity as compared to Central Gondar zone. Similarly, farming type, feed source, chicken batch management method, chicken house disinfection, litter management, presence or absence of protective footwear and clothing, and carcass management practices were all found to be significantly associated with seropositivity (*p* < 0.05) (Table 5). However, the status of house ventilation did not show a statistically significant association with seroprevalence of ILTV (*p* > 0.05) and had no confounding effect; hence, it was omitted from the final multivariable analysis in Table 5.

## 4. Discussion

The current study aimed to support the national effort to determine the status of ILTV in chicken throughout Ethiopia. A cross-sectional study was applied to determine the seroprevalence of ILTV and its associated risk factors in three zones of the Amhara region, a region with enormous chicken population [3].

We are reporting an overall seroprevalence of 59.1% (95% CI: 0.56–0.63) in the study area. Chickens may seroconvert in response to infection [26] or vaccination with ILT vaccines [27]. As we mentioned, the use ILT vaccines in Ethiopia is not officially approved by the veterinary authority. Yet, there are supposed reports of the CEO and TCO vaccines usage by private poultry farms. The use of live attenuated ILT vaccines, particularly of the CEO versions, has been linked with reversion to the virulent form causing a full-blown disease outbreak [13, 14]. Therefore, it can be speculated that the highest seroprevalence reported in this study could be the result of vaccine-induced immunity or reverted-vaccine-induced infection. Nevertheless, the finding can be considered an indication of possible circulation of the virus in the study area and warrants the need to isolate and confirm the virus from clinical cases or outbreaks indicative of ILT.

Regardless of the true cause of the seroconversion, our finding (59.1%) is in accordance with the studies of Salhi et al. [28] and Johnson et al. [29], who reported a seroprevalence of 56.25% in Algeria and 57.1% in Delmarva Peninsula, respectively.

On the other hand, our report (59.1%) is higher than that of the findings of Tesfaye et al. [18]; Roba et al. [19]; Owoade et al. [30]; Madsen et al. [31]; Aras et al. [32]; Derksen et al. [33]; Pohjola et al. [34]; Bhuiyan et al. [35]; Langeroudi et al. [36]; Uddin et al. [37]; Wunderwald and Hoop [38]; Ana et al. [39]; Baksi et al. [40]; and Shittu et al. [41], who reported a prevalence of 19.4% in Central and South Ethiopia, 54.7% in Ada'a district in Oromia region, Ethiopia, 50% in Nigeria, 49% in Maryland, USA, 42.56% in Konya region of Turkey, 46.3% in California, USA, 12% in Finland, 0.4% in selected areas of Bangladesh, 13% in broiler flocks of Iran, 17.33% in Chittagong district of Bangladesh, 28.2% in fancy breed flock of Swiss, 0.194% in Ecuador, 26.77% in India, and 1.2% in North Central Nigeria, respectively. Some other studies have reported a higher seroprevalence than ours: 67.55% in Trinidad and Tobago by Brown et al. [42]; 81.47% and 92.28% in Bangladesh by Rahman et al. [43] and Jahan et al. [44], respectively, and 89.22% in Mae Fah Luang district, Chiang Rai province of Thailand by Chukiatsiri and Pohuang [45]. Several factors can contribute to the variations observed in terms of seroprevalence of ILTV between our study and others', which include breed of chickens studied, biosecurity status of the flocks, vaccination practices in the different study areas, differences in specificity and sensitivity of tests used, and the management practices in place. In fact, the increase in poultry production density, decrease in downtime of production sites, raising of multi-age and multipurpose chicken within same geographical area, poor biosecurity, and poor vaccination methods have been linked with increased incidence of the disease [1]. In addition, vaccine virus reactivation and shedding have been reported from several commercial layers [46]. Regarding the tests, Adair et al. [47] reported that ELISA and fluorescent antibody (FA) tests have the advantages of sensitivity over serum neutralization and agar gel immunodiffusion.

In addition to detecting anti-ILTV antibodies in chickens' serum, we also assessed potential intrinsic and extrinsic risk factors that could be associated with seropositivity. In this study, seroprevalence varied significantly (*p* < 0.05) among the different study zones, with the highest seroprevalence recorded in Central Gondar zone (78.1%) followed by West Gojjam (58.6%) and South Gondar zone (40.6%). Similarly, Roba et al. [19] and Bhuiyan et al. [35] reported a significant variation in prevalence of ILTV between the different study areas they considered. Differences in management practices including biosecurity and vaccination could explain the differences. On the contrary, Salhi et al. [28] and Tesfaye et al. [18] did not observe a statistically significant difference among their study sites.

Among the host-related (intrinsic) factors, only breed showed a significant association with seroprevalence (*p* < 0.05). Local chickens had a 62% reduction in the odds of seropositivity as compared to exotic breeds (OR: 0.38, 95% CI: 0.24–0.61). Studies have shown that local breeds of chickens have better environmental adaptability and disease resistance traits than exotic breeds [48]. Other intrinsic factors such as age, sex, and production purpose of chickens exhibited no significant association with seroprevalence of ILTV (*p* > 0.05).

Among the environmental risk factors, farming type, feed source, batch management methods, chicken house disinfection, litter management, status of protective footwear and clothing, and carcass management had shown a statistically significant association with seropositivity (*p* < 0.05). Accordingly, chickens managed in an extensive system were 1.97 more likely to experience seropositivity to ILTV than chickens in the intensive farming system. In line with this, Langeroudi et al. [36] reported that backyard flocks can easily be infected due to the poor biosecurity procedures, and survived chickens would most likely become a reservoir of the virus, thus, serving as an important source of ILTV to other chickens.

In this study, the likelihood of seropositivity to ILTV was 6.53 times higher in chickens fed with locally prepared millhouse grinder leftover feed than chickens fed with commercially prepared feed. One can expect that the feed collected from the millhouse grinder left over has a higher contamination risk, becoming a vehicle for the introduction and spread of pathogens though the flock of chickens [1]. In addition, a well-nourished bird (usually the case with chickens fed on commercially prepared feed) is more immunologically competent and better able to cope with disease challenges than a poorly nourished bird [49]. However, it is unrealistic at this stage to conclude that the difference in feed source had really impacted seroprevalence among the flocks on local and commercial feed in this study. This is because seroconversion does not necessarily denote an active disease. Thus, the results should be interpreted with caution.

Management-wise, the odds of seropositivity to ILTV were 14.51 times higher in chickens kept in farms that mix different batches of chickens in one house compared to chickens managed with an *all-in-all-out* method. The introduction of new chickens, thus, of managing new and old batches of chickens mixed together is a risky action that could potentially introduce the virus to the recipient farms [50].

Similarly, the practice of inadequate disinfection has been associated with higher seropositivity of flocks. This is complementary to the well-known fact that ILTV is susceptible to the actions of organic solvents such as chloroform, ether, and oxidizing agents like 5% hydrogen peroxide [51]. Thus, farms practicing proper disinfection of their premises would have reduced infection rate.

Litter management is known to influence the occurrence of infectious diseases in a flock of chickens [52]. In our study, careless disposal of litter and the practice of using poultry litter as a fertilizer were associated with an increased and decreased seroprevalence of ILTV respectively. Consistent with this finding, Giambrone et al. [53] have provided an experimental evidence of the effectivity of in-house composting of poultry litter in reducing ILTV below the detection level using nested PCR. In any case, careful disposal of poultry litter would decrease the chance of contamination, which otherwise would serve as a source of infection for the flock [1].

Moreover, in this study, lack of usage of protective footwear and clothing and the practice of random disposal of dead chicken bodies were significantly associated with higher seroprevalence of ILTV. It is important to mention that the mechanical transmission of the ILTV through contaminated fomites has been documented [26, 54]. It has also been reported that ILTV can remain infective up to three months at room temperature while only for three weeks in buried carcass [1].

## 5. Conclusions

ILT has long been considered nonexistent and a disease of no concern in Ethiopia. This study revealed a seroprevalence of 59.1% in backyard and commercial chickens in three selected zones of Amhara region, Ethiopia. This is the first report about ILTV in the region. Thus, the finding can be regarded as an important signal that prompts further investigations about ILT in the country. The veterinary authority shall rethink the status of the disease and plan an appropriate intervention. Molecular confirmation and characterization of the virus from ILT suggestive cases should be considered to justify the use of ILT vaccines. In addition, this study has identified manageable risk factors that have significant association with seropositivity to ILTV. Application of biosecurity procedures would therefore have an utmost impact on the control of the disease.

## Figures and Tables

**Table 1:** Summary of potential risk factors and their respective frequency.

Potential factors	Categories	Frequency (%)
Zone	Central Gondar	256 (33.3)
	South Gondar	256 (33.3)
	West Gojjam	256 (33.3)
Breed	Local	415 (54)
	Exotic	353 (46)
Sex	Male	219 (28.5)
	Female	549 (71.5)
Farm type	Intensive	213 (27.7)
	Extensive	555 (72.3)
Production type	Layer	445 (57.9)
	Broiler	171 (22.3)
	Dual	153 (19.8)
Age	Young (3 week-21 week)	201 (26.2)
	Adult (>21 week)	567 (73.8)
Feed source	Locally prepared	119 (15.5)
	Commercially prepared	649 (84.5)
Batch management	All in-All out	130 (16.9)
	Different batches in one house	638 (83.1)
Ventilation	Well ventilated	584 (76)
	Partially ventilated	184 (24)
Litter management	Buried	69 (9)
	Used as fertilizer	426 (55.5)
	Accumulate near space	273 (35.5)
House disinfection	Disinfected after each batch	226 (29.4)
	No disinfection	542 (70.6)
Carcass management	Burying or burning	131 (17.1)
	Throwing to nearby place	637 (82.9)
Protective footwear and clothing	Available	105 (13.7)
	Not available	663 (86.3)
Total		768 (100)

**Table 2:** Seroprevalence of ILTV in chickens by study zones.

Study zones	Number examined	Indirect ELISA result		Prevalence (%)
		Positive	Negative	
Central Gondar	256	200	56	78.1
South Gondar	256	104	152	40.6
West Gojjam	256	150	106	58.6
Total	768	454	314	59.1

**Table 3:** Univariable and multivariable mixed-effect logistic regression analysis of host-related risk factors with ILTV seroprevalence.

Variables Category	No. of examined	No. of positive	Prevalence % (95% CI)	Univariable		Multivariable	
				COR (95% CI)	*pvalue*	AOR (95% CI)	*pvalue*
Breed							
Exotic	415	257	61.9 (0.57–0.67)	RF			
Local	353	197	55.8 (0.50–0.61)	0.78 (0.58–1.04)	0.086	0.38 (0.24–0.61)	≤0.001^*∗*^
Sex							
Male	219	128	58.5 (0.52–0.65)	RF			
Female	549	326	59.4 (0.55–0.64)	1.04 (0.76–1.43)	0.812		
Age							
Young	201	106	52.7 (0.46–0.60)	RF			
Adult	567	348	61.4 (0.57–0.65)	1.42 (1.03–1.97)	0.033		
Production purpose							
Layer	445	265	69.6 (0.55–0.64)	RF			
Broiler	171	93	54.4 (0.47–.62)	0.81 (0.57–1.16)	0.263		
Dual	152	96	63.2 (0.55–0.71)	1.16 (0.79–1.70)	0.432		

COR: crude odds ratio AOR: adjusted odds ratio, 95% CI: 95% confidence interval. *p* value of <0.05 was considered statistically significant, and *p* value of ≤0.001^*∗*^ was considered strong statistical significance, RF: reference factor.

**Table 4:** Univariable and multivariable analysis of seroprevalence in relation to study zones.

Study zones	Sample size	No. of positive	Prevalence % (95% CI)	Univariable		Multivariable	
				COR (95% CI)	*pvalue*	AOR (95% CI)	*pvalue*
Central Gondar	256	200	78.1 (0.73–0.83)	RF			
South Gondar	256	104	40.6 (0.35–0.47)	0.19 (0.13–0.28)	0.001	0.49 (0.29–0.86)	0.010
West Gojjam	256	150	58.6 (0.52–0.65)	0.40 (0.27–0.58)	0.001	0.59 (0.34–0.97)	0.002

**Table 5:** Univariable and multivariable mixed-effect logistic regression analysis of environmental risk factors with ILTV seroprevalence.

Variables category	Sample size	No. of positive	Prevalence % (95% CI)	Univariable		Multivariable	
				COR (95% CI)	*p* value	AOR (95% CI)	*p* value
Farming type							
Intensive	213	97	45.5 (0.39–0.53)	RF			
Extensive	555	357	64.3 (0.60–0.68)	2.16 (1.56–2.97)	≤0.001^*∗*^	1.97(1.01–5.02)	≤0.001^*∗*^
Feed source							
Commercially prepared	119	55	46.2 (0.37–0.56)	RF			
Locally prepared	649	399	61.5 (0.58–0.65)	1.86 (1.25–2.75)	0.002	6.53 (1.77–24.04)	0.005
Batch management							
All-in-All-out	130	63	48.5 (0.57–0.65)	RF			
Different batches in one house	638	391	61.3 (0.57–0.65)	1.68 (1.15–2.46)	0.007	14.51(3.35–62.77)	≤0.001^*∗*^
Ventilation							
Well ventilated	584	354	60.6 (0.57–0.65)	RF			
Partially ventilated	184	100	54.3 (0.47–0.62)	0.77 (0.55–1.08)	0.132		
House disinfection							
Disinfection after each batch of chicken	226	63	27.9 (0.22–0.34)	RF			
No disinfection	542	391	72.1 (0.68–0.76)	6.70 (4.74–9.47)	0.037	11.05(4.09–47.95)	≤0.001^*∗*^
Litter management							
Buried	69	43	62.3 (0.50–0.74)	RF			
Used as fertilizer	426	225	52.8 (0.48–0.58)	0.68 (0.40–1.14)	0.143	0.20 (0.05–0.76)	0.018
Accumulate to the nearby free space	273	186	68.1 (0.62–0.74)	1.29 (0.75–2.24)	0.232	1.62 (1.08–4.37)	≤0.001^*∗*^
Protective footwear and clothing							
Available	105	19	18.1 (0.11–0.27)	RF			
Not available	663	435	65.6 (0.62–0.69)	8.64 (5.12–14.56)	≤0.001	20.85 (5.40–80.45)	≤0.001
Carcass management							
Burying or burning	131	37	28.2 (0.21–0.37)	RF			
Throwing to nearby place	637	417	65.5 (0.62–0.69)	4.82 (3.18–7.29)	≤0.001	13.25 (5.31–52.34)	0.001

COR: crude odds ratio, AOR: adjusted odds ratio, 95% CI: 95% confidence interval, *p* value of <0.05 was considered statistically significant, *p* value of ≤0.001^*∗*^ was considered strong statistical significance, RF: reference factor.

## Data Availability

Data will be made available upon request to the primary author.
